# Assessing the impact of industrial intelligence on urban carbon emission performance: Evidence from China

**DOI:** 10.1016/j.heliyon.2024.e30144

**Published:** 2024-04-23

**Authors:** Chenglin Tu, Chuanxiang Zang, Anqi Wu, Hongyu Long, Chenyang Yu, Yuqing Liu

**Affiliations:** a School of Management, Guangzhou University, University Town Outer Ring West Road 230, 510006, Guangzhou, China; b Academy of Guangzhou Development, Guangzhou University, University Town Outer Ring West Road 230, 510006, Guangzhou, China; c NTU Entrepreneurship Academy, Nanyang Technological University, 50 Nanyang Ave, 639798, Singapore; d Innovation, Policy and Entrepreneurship Thrust, The Hong Kong University of Science and Technology, Guangzhou, 511455, China; e School of Humanities, Guangzhou University, Guangzhou, 510006, China

**Keywords:** Industrial intelligence, Carbon emission performance (CEP), Spatial spillover effect, Producer service agglomeration

## Abstract

With the growing emphasis on sustainable development, there has been increasing attention given to measures aimed at promoting environmental improvements and reducing carbon emissions, including the adoption of intelligent industry. Recent studies have analyzed the influence of industrial intelligence on urban carbon emission performance while ignore the spatial spillover effects and lack in-depth discussion of the mechanisms, which reduces the reliability of the assessment of industrial intelligence's impact on carbon emission performance. To address this issue, the paper examines direct effect, spatial spillover effects, and mechanisms, utilizing a balanced panel data from 2008 to 2019 for 238 Chinese cities. The findings reveal that a 1 % improvement in industrial intelligence results in a 2.747 % enhancement of local carbon emission performance. Moreover, through the spatial spillover analysis, we determined that industrial intelligence has a notable negative impact on the carbon emission performance of surrounding areas. The mechanism analysis demonstrated that industrial intelligence affects the carbon emission performance of local and neighboring areas by influencing the agglomeration of productive services. Furthermore, our study illustrates that the industrial intelligence's enhancement effect on carbon emission performance shows more significantly in eastern, resource-dependent, and ordinary prefecture-level cities. Finally, endogeneity and robustness tests conducted yielded consistent conclusions. Our study provides a new perspective on industrial intelligence's carbon reduction effect and contributes to the development of policies related to industrial upgrading and green development.

## 1 Introduction

### 1.1 Research background

In recent years, the growing demand for global economic growth has heightened the importance of pressing concerns like global warming and pollution [[1], [2], [4], [5], [6], [7], [3]]. Such environmental degradation poses great challenges to sustainable development in human society. The report by the International Energy Agency (IEA) titled “Global Energy Review: CO_2_ Emissions 2021” has revealed an alarming trend of global carbon emissions reaching pre-pandemic levels. According to the report, in 2021, global CO_2_ emissions increased by 6 %, reaching a significant 36.3 billion tons, with China playing a significant role in driving this trend [8,9]. It's important to highlight that China, as the only major economy that experienced growth during the COVID-19 pandemic, saw its CO_2_ emissions increase by 750 million tons in just two years. In comparison, the rest of the world experienced a total decline in emissions of 570 million tons. However, China is a proactive signatory of the Paris Agreement, pledging to work in collaboration with other participating nations to achieve the shared goal of capping the increase in global average temperature to no more than 2 °C above pre-industrial levels [10,11]. Achieving the goal of harmonizing economic growth and reducing carbon emissions necessitates the adoption of effective strategies for optimizing carbon efficiency [[12], [13], [14]]. Numerous countries have endeavored to adopt diverse approaches towards enhancing carbon emission performance (CEP) or efficiency to support sustainable development. In particular, China has implemented policies such as the carbon regulation policy and “low carbon city pilots” regulation to increase carbon emission performance [[15], [16], [17]]. Some countries like The United States, as environmental pollution issues are effectively controlled, efforts have been made to enhance carbon emission performance through the introduction of the Clean Energy and Energy Security Act [[18], [19], [20]]. Additional nations have similarly implemented policies focused on carbon emission performance [[21], [22], [23]]. Hence, improving carbon efficiency and performance remains a compelling aspect of sustainability (Behzadi et al., 2022; [24]).

Owing to advancements in electronics and intelligent technology, industrial intelligence have become increasingly prevalent across several nations [1,25]. As of 2021, the global average density of industrial robots has surged to 126 units per 10,000 workers, which represents a threefold increase from 2015's corresponding figures (66 robots per 10,000 workers). The discussion on whether industrial intelligence can provide new impetus for the enhancement of carbon emission performance has emerged as a focal point of scholarly discourse. Various studies have indicated that the implementation of industrial intelligence can be a viable means of improving CEP and energy efficiency. For example, Liu et al. [26] studied AI's impact on carbon's intensity based on the database from Chinese industrial sector and found that AI had a significant reduction in carbon intensity. Similar findings were found in the study of Li et al. [27]. They used a sample consisting of 35 countries to assess the emission reduction effects of industrial intelligence and concluded that industrial intelligence can significantly improve energy efficiency through optimization of factor structure and technological innovation. However, some scholars argued the opposite. They supposed that digitization and intelligence are driving economic growth while increasing carbon emission and reducing carbon efficiency. Wang et al. [28] investigated correlation between carbon emission efficiency and internet economy by analyzing panel provincial data from 2006 to 2017 in China. Their analysis revealed an obvious adverse effect of the internet economy on carbon emission efficiency in southern China. Also, the study by Kopp and Lange [29] revealed that the rebound effect associated with the construction and maintenance of intricate digital infrastructure can exert a substantial detrimental impact on the environment and, by extension, carbon emission performance. Based on the available literature, it is apparent that a consensus has yet to be reached regarding industrial intelligence's effect on CEP. Our paper posits that current research has largely overlooked industrial intelligence's spatial spillover effect on CEP. While industrial intelligence can enhance local CEP by promoting industrial transformation, upgrading, and relocation away from traditional enterprises, it may also compel neighboring regions to accommodate low-end enterprises and traditional industrial workers, thereby compromising the CEP of such regions. These findings suggest that industrial intelligence, despite improving local CEP, may also generate negative spillover effects on neighboring regions. Neglecting such effects could potentially undermine the validity of the research findings. Hence, it is imperative to start comprehensive investigations on industrial intelligence's impact on CEP utilizing spatial econometric models.

To fill the research gap mentioned above, this paper employs Stochastic Frontier Analysis (SFA) method to estimate CEP data using Chinese prefecture-level-cities spatial panel data [30,31]. Furthermore, the paper examines potential spatial spillover effects of industrial intelligence on CEP by incorporating spatial measures into the analysis [32,33]. It also delves into the underlying mechanisms through which industrial intelligence affects CEP and subjects them to empirical testing. Additionally, this paper investigates industrial intelligence's impact on CEP across different regions, varying levels of resource dependency, and different administrative levels of cities. Finally, a series of rigorousness assessments are performed to ensure the soundness and dependability of the research findings.

### 1.2 Contribution of this paper

The paper makes several contributions to the existing research. Firstly, it investigates industrial intelligence's spatial spillover effects on CEP using spatial econometric methods. Prior research has focused on industrial intelligence's direct impact on CEP, without considering differences in industrial intelligence's impact on neighboring and local areas' CEP. Therefore, the present study offers a more comprehensive assessment of industrial intelligence's impact on CEP, thus providing novel insights into the development of regional carbon emission reduction policies. Second, our article delves into intrinsic mechanism of industrial intelligence's spatial spillover effect on CEP. While existing studies have explored direct impact mechanisms about industrial intelligence use on carbon efficiency and energy performance, they have yet to conduct a mechanism analysis for spatial spillover effects of industrial intelligence. Thus, it is crucial to investigate comprehensively the mechanisms that lead to spatial spillover effects of industrial intelligence on CEP. Our findings can offer new perspectives for policy optimization to minimize efficiency losses in industrial intelligence process. Third, the paper provides an in-depth analysis on heterogeneous impact of industrial intelligence on CEP of cities with different economic development, resource dependence, and administrative levels. Regional disparities in development, such as variations in economic, resource, and administrative levels, are ubiquitous across countries worldwide. Ignoring such heterogeneity can result in either overestimation or underestimation of industrial intelligence's emission reduction effects and undermine effectiveness of policy implementation. Thus, it is imperative to explore industrial intelligence's impact on CEP of cities with diverse characteristics to identify and address regional disparities and optimize policy implementation.

### 1.3 Structure of this paper

The next parts of paper are structured as follows: Section two presents the literature review and research hypothesis; Section three present the methods and data; Section four outlines the results; Section five presents the heterogeneity analysis; Section six presents the mechanism analysis; Section seven concludes with recommendations.

## 2 Literature review and research hypothesis

### 2.1 Definition of the concept

In that section, the paper begins by defining and explaining several foundational concepts involved in the study to build the theoretical foundation of this study. The first concept that needs to be defined is carbon emissions. In a broad sense, carbon emissions refer to the amount of carbon dioxide emitted from natural and human activities [34]. In this study, carbon emissions specifically refer to carbon dioxide emissions from human activities at the urban geographic scale. The second concept that needs to be defined is sustainability. A large body of literature shows that economic development is inevitably accompanied by a rapid increase in carbon emissions [35]. Increased carbon emissions exacerbate climate change, thereby increasing the risk of natural disasters and threatening the human habitat [36]. In the context of this study, sustainability refers to the realization of economic development within the context of manageable climate change risks. Finally, industrial intelligence is also a key concept covered in this paper. In this study, industrial intelligence refers to the extent to which machines replace humans in production activities [37]. A higher level of this degree indicates a higher level of industrial intelligence.

### 2.2 Literature review on urban carbon emission performance

This section offers a thorough examination of the pertinent literature pertaining to CEP, with a focus on two main aspects: first, the definition and measurement of CEP; and second, the factors that affect CEP.

Scholars often characterize CEP as the effectiveness of utilizing carbon emissions to generate output, with per unit economic output of carbon emissions being a common metric of measurement (Dong et al., 2022). A greater magnitude of this parameter corresponds to a heightened CEP, signifying relatively decreased carbon emissions for each unit of economic output. Nevertheless, this approach fails to consider the impact of various other factors on CEP. For instance, through different mechanisms, government expenditures and total industrial output can affect the performance of carbon emissions to varying degrees. Therefore, to address these limitations, some scholars have employed the Stochastic Frontier Analysis method (SFA) and Data Envelopment Analysis method (DEA) [6,38]. The DEA method is capable of measuring CEP based on multiple inputs and outputs, but it requires no prior assumption about the functional form of the production function, rendering it vulnerable to stochastic errors. By contrast, the SFA method offers the flexibility of setting model variables to align with the study context and incorporates stochastic errors. However, many studies are conducted based on large samples at the prefecture or even enterprise level which require consideration of heterogeneity, while traditional DEA and SFA methods are not capable of accounting for this heterogeneity. To account for the issue of heterogeneity, Kumbhakar et al. [39] introduced a developed SFA methodology which incorporates time-invariant and time-varying characteristics, as well as heterogeneity of urbans in residuals. This approach has been demonstrated to yield a more all-encompassing assessment of performance in the presence of heterogeneity. Thus, we utilize the refined SFA method to measure CEP in our study.

Numerous studies have investigated the determinants of regional CEP, indicating that factors such as GDP per capita, industrial structure, government governance, financial development, and foreign investment exert varying degrees of influence on CEP [[40], [41], [42], [43], [44]]. Some scholars have focused on the aggregate output of the region. Wang and Li [45] investigated different factors' effects on carbon emissions including employment rate, urbanization, and GDP per capita. They concluded that the increase of GDP per capita is contributes to carbon emission intensity's reduction and carbon emission efficiency's improvement. However, some scholars reached the opposite conclusion. Adeleye et al. [46] conducted an empirical investigation on relationship between carbon emissions and income per capita using samples from African countries. The results they obtained demonstrated a positive correlation between carbon emissions and both income and economic growth, which is in accordance with the conclusions drawn by Phong (2018). In addition to GDP per capita, which has been identified as a significant influence on carbon emissions efficiency across various areas, industrial structure is also a crucial factor. In the study by Wang et al. [47], an analysis was conducted on the estimation of carbon emission efficiency and reduction potential in various Chinese provinces. Additionally, the study investigated the indirect impacts of natural resource abundance on emission efficiency. These authors concluded that the advancement of industrial structure can greatly enhance CEP. Sun and Huang [48] suggested that current industrial structure may impede carbon efficiency's development. Additionally, government governance has been shown to impact CEP. Some scholars found that governments can effectively improve carbon efficiency by intervening in carbon emissions through the development of relevant policies and programs [40,48]. Yan et al. [43] conducted a sector-specific analysis on government intervention's impact of producer services agglomeration's effect on CO_2_ emission reduction. Their findings indicate that it would be beneficial for the government to increase its support to producer service industries and undertake appropriate interventions to enhance carbon emission efficiency. Meanwhile, the degree of regional financial development is also a significant factor that influences carbon emissions and performance. The study conducted by Shahbaz et al. [49] provides evidence in favor of the notion that financial development is conducive to reducing carbon emission and enhancing environment's improvement. Furthermore, Acheampong et al. [41] conducted a nuanced investigation into financial market development's impact on carbon intensity across different stages of national development. They found that while the overall development of financial markets decreases carbon intensity in developed and emerging economies, the effect is reversed in frontier economies. Finally, this paper argues that FDI also has impacts on regional CEP. Shahbaz et al. [49] arrived at significant positive effects of FDI on carbon emission based on their analysis of a French sample. Zhang and Zhang [50] corroborated this finding through their study of the Chinese sample. In a similar vein, Khan et al. [44] discovered that FDI and trade contributed to increased carbon emissions in developing nations but reduced them in “Belt and Road” countries.

### 2.3 Literature review on industrial intelligence

In this section, this paper provides a synthesis of the literature on industrial intelligence from three perspectives: its effects on the technology sector, labor market, and resources and environment.

On one hand, a considerable body of literature has examined industrial intelligence's impact on technology sector and found that the use of industrial intelligence can have a substantial positive effect on technology and productivity. For example, Kromann et al. [51] conducted a study on automation's impact on total factor productivity using industry-level panel data from nine countries. Their findings indicated that intensive industrial intelligence has a notable positive impact on total factor productivity. These results are consistent with conclusions drawn from an article conducted by Jung and Lim [52] and Philippe et al. [53] on the same topic. On the other hand, researchers have also directed their attention towards industrial intelligence's influence on labor market. Acemoglu and Restrepo [54] investigated on the effects of the increased industrial intelligence on the local labor market using a sample from the United States dating from 1990 to 2007. Their findings suggest that for each additional industrial robot per 1000 workers, the ratio of employment to total population would decrease by approximately 0.18–0.34 percent, and the wage level would decrease by 0.25–0.5 percent. In contrast, some scholars hold an optimistic view regarding the potential positive effects of industrial intelligence on labor markets [55,56]. Webb [55] introduced a novel approach to predict the effects of technological advancements on the labor market. By constructing a measure of task automation based on the overlap between job task descriptions and patent texts, he investigated the impact of artificial intelligence on wage inequality. His findings suggest that if the historical pattern of long-term substitution persists, AI could potentially reduce wage inequality by 90:10. Besides the labor market, industrial intelligence also has a significant impact on resources and environment. Chen et al. [57] conducted a study examining the association between industrial intelligence utilization and ecological footprint using a dataset spanning 72 countries from 1993 to 2019. Their research revealed that the industrial intelligence can contribute to reducing ecological footprint through three main channels. Moreover, they observed that impact of industrial intelligence on reducing ecological footprint appears more obviously in highly developed countries with higher levels of human capital. Huang et al. [58] utilized a PSM-DID methodology to examine the connection between user innovation rights and energy efficiency based on data collected from Chinese firms spanning the period 2001 to 2012. Their findings suggest that industrial intelligence in production significantly improved energy efficiency, with productivity gains being the primary driver of the improvement, rather than a reduction in total energy consumption. In an independent investigation, Luan et al. [59] scrutinized the effect of industrial intelligence utilization on air quality, with mediation through energy consumption and moderation through population density. This study made use of a longitudinal dataset consisting of 74 countries and regions, spanning the period from 1993 to 2019. Their results indicate that as industrial intelligence increased productivity and energy efficiency, it also stimulated production and consumption, ultimately leading to increased overall energy consumption, air pollution, and climate warming.

### 2.4 Research hypothesis

Within this part, the current investigation will examine the influence of industrial intelligence on CEP through an analysis of the extant literature and an exploration of the underlying mechanisms that have been proposed to account for impact of industrial intelligence on CEP.

Firstly, existing studies primarily investigate impact of industrial intelligence on CEP through various sample analyses (Li et al., 2022 [60]; Liu et al., 2022). Liu et al. (2022) developed a stochastic impact model that considers population, affluence, and technology regressions to examine AI's impact on carbon intensity through a Chinese database. Their findings indicated that AI is associated with a decrease in carbon intensity and is accompanied by a noteworthy degree of industrial heterogeneity. Similarly, utilizing EKC model, Li et al. [27] conducted an empirical evaluation of industrial intelligence adoption's impact on carbon reduction. Based on data gathered from a sample of 35 countries covering the period from 1993 to 2017, they observed that industrial intelligence results in productivity gains, technological innovation in production, and optimization of factor structure, leading to an increased number in energy efficiency and a reduction in carbon intensity.

Secondly, the article argues that the impact of industrial intelligence on CEP is not confined to local settings but may also exhibit spatial spillover effects. Although Li et al. [27] and Liu et al. [26] conclude that industrial intelligence can improve CEP, they both ignore the spatial spillover effect. Given potential spatial correlation in the distribution of industrial intelligence, its impact on both local and neighboring CO_2_ emission performance may exhibit variations. According to the “machine takes human's place” theory [61], the spread of industrial intelligence will cause a reduction in local traditional industrial jobs. Simultaneously, the rise in unemployment within local traditional industries can lead to a rise in employees' number within low-end industries in neighboring regions. Since the impact of the talent flow on regional industrial structure, as highlighted by Zhou et al. [62], it can be argued that a more optimized local industrial structure is likely to contribute to improved CEP. Conversely, it has also been noted that adjacent areas tend to witness a reduction in CEP because of the elevation in the proportion of traditional industries within their industrial framework. As such, this paper puts forward hypothesis 1.

H1 industrial intelligence can enhance local CEP and reduce CEP in neighboring places.

Finally, the paper contends that the spatial spillover effect of industrial intelligence on carbon emissions can be attributed to two primary mechanisms. The current industrial intelligence is mainly focused on high-end industries, while some lower-end traditional industries have not completed intelligent transformation, and their productivity and profit situation will be insufficient to support enterprises to join the competition [63,64]. Hence, some low-end traditional industries will be moved outside the region, while high-end industries will remain locally. As high-end industries and manufacturing sectors typically require financial, economic, and cultural support, their presence can stimulate the growth of local producer service industries and attract neighboring firms in the same sector to the area [65]. In general, the local production service agglomeration level will be further enhanced due to the improvement of industrial intelligence and proportion of high-end industries. Neighboring regions may attract traditional industries that have been transferred from the local area, while simultaneously losing some of their producer service industries to the local area. As a result, the concentration of the production service industry in adjacent regions is anticipated to decrease. Several empirical studies [14,66,67] have demonstrated that the concentration of producer service industries can positively impact carbon efficiency. It is reasonable to propose that industrial intelligence has the potential to improve local carbon efficiency by promoting the concentration of producer service industries. Thus, it can be inferred that the enhancement of local agglomeration of producer service industries due to industrial intelligence can potentially result in improving local carbon emission efficiency. Conversely, the decrease in the concentration of producer services in adjacent regions resulting from industrial intelligence result in a deterioration of their carbon emission efficiency. Consequently, this paper puts forward the hypothesis 2.

H2 industrial intelligence can improve local CEP by increasing the level of local producer service agglomeration and reduce neighboring CEP by decreasing the level of neighboring producer service agglomeration.

## 3 Methods and data

### 3.1 Empirical model

This study employs spatial econometrics to examine the effect of industrial intelligence on CEP. A plethora of previous research endeavors have utilized various spatial econometric techniques such as spatial lag models (SLM), spatial error models (SEM), and spatial Durbin models (SDM) to examine econometric issues with spatial relevance [[68], [69], [70]].The spatial lag model (SLM) entails incorporating the dependent variable that is lagged spatially into the model, while the spatial error model (SEM) entails incorporating the error term that is lagged spatially into the model. On the other hand, the spatial Durbin model (SDM) entails incorporating both the dependent and independent variables that are lagged spatially into the model [71]. Furthermore, this research utilizes the geographic distance weight matrix as the spatial weight matrix in the model, which is formulated as formula (1).

(1) $$W_{i,j}=\left\{ \begin{array}{c} e^{-\alpha d_{ij}},i\neq j\\ 0,i=j \end{array}\right.$$

where $W_{i,j}$ represents a matrix of spatial weights; α represents the geographical distance coefficient; $d_{ij}$ represents the geographical distance between i and j. The diagonal elements are all 0 in this matrix.

The SLM method is an extension of the OLS model, where the dependent variable includes a spatial lag term. This means that the dependent variable in the SLM method is not only related to the local independent variable, but also to the dependent variable in the neighboring areas [72]. The SLM method can be represented in its fundamental form as formula (2):

(2) $$Y_{it}=\alpha +\delta \sum_{j=1}^{n}W_{ij}Y_{it}+\beta X_{it}+\varepsilon_{it}\;\varepsilon_{it}∼N\left(0,\sigma^{2}I\right)$$

where $Y_{it}$ represents the CEP; $X_{it}$ represents industrial intelligence; $\delta \sum_{j=1}^{n}W_{ij}Y_{it}$ represents the spatial lag term in CEP.

The SLM method considers the potential effect of dependent variables in neighboring regions on the dependent variable in the focal area by incorporating a spatial lag term into the econometric equation. On the other hand, the SEM method addresses the spatial dependence of unobserved factors that could influence the independent variables in the focal area due to their proximity to other regions. These factors are introduced into the econometric equation as a spatial error term [73]. The SEM method can be represented in its fundamental form as formula (3):

(3) $$Y_{it}=\alpha +\beta X_{it}+\varepsilon_{it}$$

(4) $$\varepsilon_{it}=\lambda W_{it}\varepsilon +\mu \;\mu ∼N\left(0,\sigma^{2}I\right)$$

where $Y_{it}$ represents the CEP; $X_{it}$ denotes industrial intelligence; λ in formula (4) represents the coefficient estimate of the spatial autocorrelation error term.; μ represents the error term in the model.

As the spatial error effect and spatial lag effect are commonly observed together, a novel model that integrates these two effects has been developed, known as the spatial Durbin model (SDM) [71]. The SDM method is advantageous for two key reasons. Firstly, it can produce unbiased coefficient estimates if the true data generation process incorporates both spatial lags and spatial errors. Secondly, the model is free from any a priori restrictions on the magnitude of potential spatial spillover effects. SDM method can be represented in its fundamental form as formula (5):

$$Y_{it}=\alpha +\delta \sum_{j=1}^{n}W_{ij}Y_{it}+\beta X_{it}+\xi \sum_{j=1}^{n}W_{ij}X_{it}+\lambda {Con}_{it}$$

(5) $$+\tau \sum_{j=1}^{n}W_{ij}{Con}_{it}+\varepsilon_{it}\;\varepsilon_{it}∼N\left(0,\sigma^{2}I\right)$$

where $Y_{it}$ represents CEP; $X_{it}$ represents industrial intelligence; ${Con}_{it}$ represents control variables; $\sum_{j=1}^{n}W_{ij}Y_{it}$ represents the spatial lag term in CEP; $\sum_{j=1}^{n}W_{ij}{Con}_{it}$ represents the spatial lag term in control variables; $\sum_{j=1}^{n}W_{ij}X_{it}$ represents the spatial lag of industrial intelligence.

According to other scholars [74], when spatial models display spatial hysteresis, techniques of point estimation for analyzing spatial spillover effects may result in bias. In these scenarios, calculus-based methods that differentiate between direct and indirect effects can be utilized to calculate the total effect. The SDM method can be re-expressed as formula (6):

(6) $$Y_{t}={\left(1-\delta W\right)}^{-1}\left(\beta X_{t}+\gamma WX_{t}\right)+{\left(1-\delta W\right)}^{-1}\varepsilon_{t}$$

If we take the *k* independent variables as independent variables in the above equation, the outcome can be represented as a partial differential matrix as formula (7):

(7) $${\left[\begin{array}{ccc} \frac{\partial Y}{\partial X_{1k}} & \cdots & \frac{\partial Y}{\partial X_{NK}} \end{array}\right]}_{t}={\left(1-\delta W\right)}^{-1}\left[\begin{array}{cccc} \begin{array}{c} \beta_{k}\\ W_{21}\lambda_{k}\\ \vdots \\ W_{N1}\lambda_{k} \end{array} & \begin{array}{c} W_{12}\lambda_{k}\\ \beta_{k}\\ \vdots \\ W_{N2}\lambda_{k} \end{array} & \begin{array}{c} \cdots \\ \cdots \\ \ddots \\ \cdots \end{array} & \begin{array}{c} W_{1N}\lambda_{k}\\ W_{2N}\lambda_{k}\\ \vdots \\ \beta_{k} \end{array} \end{array}\right]$$

The partial differential matrix demonstrates that the diagonal and off-diagonal elements represent the direct and indirect effects of independent variable changes in the focal region and neighboring regions, respectively.

### 3.2 Variable selection

#### 3.2.1 Dependent variable

This study adopts CEP as the dependent variable. There are many methods to measure CEP and investigate the driving factors [75,76]. Among them, the SFA model is commonly employed, which involves decomposing the residuals post-parameter regression to derive the CEP [77]. The conventional SFA model is limited in its ability to disentangle the unobserved heterogeneity present in the residuals. As a result, this can pose challenges in effectively eliminating the influence of inefficiency factors from the regression residuals, thus impairing the reliability of the results [78]. To compute CEP, we utilize Kumbhakar's expanded SFA method. The proposed model introduces stochastic noise to the efficiency analysis, thus mitigating the issue of unobserved heterogeneity in carbon emission efficiency estimates. Furthermore, the model distinguishes between time-invariant, time-varying and individual effects in model's residual, which strengthens reliabilities. Extended SFA model is given below:

(8) $${CE}_{i,t}=\beta_{0}+f\left(X_{i,t};\beta \right)+\mu_{it}+\lambda_{i}-\tau_{it}-\gamma_{i}$$

(9) $$PCEP_{i}=\exp\left(-{\hat{\gamma }}_{i}\right)$$

(10) $$RCEP_{i,t}=\exp\left(-{\hat{\tau }}_{it}\right)$$

(11) $$CEP_{i,t}=PCEP_{i}\times RCEP_{i,t}$$

where carbon emission in city i in year t is represented by ${CE}_{i,t}$, where $f\left(X_{i,t};\beta \right)$ represents carbon emission's random frontier function with $X_{i,t}$ as the output factor and β as the regression coefficient. formula (8) includes the regression error term $\mu_{it}$, urban effect $\lambda_{i}$, and inefficiencies of continuous and residual carbon emission $\tau_{it}\geq 0$ and $\gamma_{i}\geq 0$ respectively, which satisfy the distribution requirements of $\mu_{it}∼N\left(0,\sigma_{u}^{2}\right)$, $\lambda_{i}∼N\left(0,\sigma_{\lambda }^{2}\right)$, $\tau_{it}∼N^{+}\left(0,\sigma_{\tau }^{2}\right)$, $\gamma_{i}∼N^{+}\left(0,\sigma_{\gamma }^{2}\right)$. The PCEP_i_ in the above formula (9) refers to the constant carbon emission performance, while the RCEP_i,t_ in formula (10) represents the time-varying and city-specific residual carbon emission performance. In formula (11), Multiplying the constant carbon emission performance (PCEP) by the residual carbon emission performance (RCEP) yields the overall carbon emission performance (CEP).

#### 3.2.2 Independent variable

This paper adopts the industrial intelligence level (IINT) as the independent variable. Meanwhile, this paper adopts the use of industrial robots to evaluate industrial intelligence in cities. Acemoglu and Restrepo [54] combined three indicators (Industrial employment structure in cities, Number of urban workforces and Number of industrial robots at the industry level) to create a city-level indicator of IINT in formula (12):

(12) $${IINT}_{c,t}=\sum_{j}\frac{{Emp}_{j,c,t}}{\sum_{j}{Emp}_{j,c,t}}\times \frac{{Robots}_{j,t}}{{Labor}_{c,t}}$$

where c represents city, j represents industry, and t represents time. ${Robots}_{j,t}$ represents the number of robots used in 14 manufacturing industry sub-sectors in China from 2008 to 2019. The variable ${Emp}_{j,c,t}$ denotes the number of employees at the city-industry level, while ${Labor}_{c,t}$ represents the number of age-appropriate labor force in a city. Thus, the numerator represents the number of robots used by city c in industry j in year t. The denominator represents the total employed population of city c in year t. By adding up the data for each industry within the city in the numerator and dividing it by the total employed population in the city, the IINT of city c in year t is obtained.

#### 3.2.3 Control variable

The control variables selected in this paper include industrial structure (*is*), the natural logarithm of GDP per capita (*lnrgdp*), financial development level (*fin*), foreign direct investment level (*fdi*) and government governance level (*gov*) [[42], [43], [44], [45], [46],^79^,^80^]. Table 1 presents the detailed measurement of each variable.

**Table 1:** Definition of selected variable.

Variable type	Symbol	Variable detail	Method
Dependent variable	cep	Carbon emission performance	Calculation based on expanded SFA model
Independent variables	IINT	Industrial intelligence	Logarithm of robots used/installed per 10,000 people
Control variables	lnrgdp	Development of economy	Logarithm of GDP per capita
	is	Industry structure	Value added of tertiary industry/value added of secondary industry
	gov	Intervention of Government	Government expenditure/GDP
	fin	Development of finance	Deposit and loan balance of financial institutions/GDP
	fdi	Level of foreign investment	Actual amount of foreign capital utilized/GDP
Variables in KLH-SFA	∅ lngdp	Economic aggregate	Logarithm of GDP
	∅ lnpop	Total population	Logarithm of population
	∅ lnge	Government expenditure	Logarithm of government expenditure
	∅ lnind	Total industrial output	Logarithm of total industrial output

### 3.3 Data

To comply with the spatial panel model requirements, this study adopts a balanced panel data approach by analyzing a sample of 238 Chinese cities from 2008 to 2019. The dataset comprises 2856 observations, including carbon emission data obtained from the open-access Spatial Grid Monthly Anthropogenic Carbon Emissions Dataset (ODIAC) [81]. The ODIAC database provides detailed carbon information at a resolution of 1 km × 1 km, which is combined to create a carbon emission dataset for prefecture-level cities in China. In this paper, 2008 to 2019 is selected as the sample period, and 2020 and updated data are not included for the following two main reasons. First, the carbon emissions dataset is only updated to 2019, so there are limitations in the availability of carbon emissions data for 2020 and beyond. Second, due to the outbreak of COVID-19, some of the statistical indicators for 2020 to 2022 are missing and may not be improved until the next few years. In this paper, a large number of city-level control variables are selected, and the spatial econometric model must use balanced panel data. Including data from 2020 and beyond would result in a large sample loss and reduce the reliability of the estimates. Since this paper focuses on the effect of industrial intelligence on the urban carbon emission performance, this objective rule is not significantly altered by the lack of a two-to three-year sample period. Therefore, the findings of this paper are reliable even if samples from 2020 and beyond are not included. Summarizing the above considerations, this paper finally selects the panel data of 238 prefecture-level cities between 2008 and 2019 as the research sample. To control for city characteristics, this paper includes several variables sourced from China City Statistical Yearbook and CNRDS Platform. To provide a better understanding of the dataset used in this study, Table 2 presents the descriptive statistics for each variable included in the analysis.

**Table 2:** Descriptive statistics.

Variable	Obs	Mean	Std	Min	Median	Max	Skewness	Kurtosis
cep	2856	0.497	0.169	0.071	0.505	0.818	−0.355	2.232
IINT	2856	0.171	0.377	0.000	0.000	1.000	1.746	4.047
lnrgdp	2856	0.481	0.099	0.117	0.482	0.851	−0.258	5.283
is	2856	0.174	0.079	0.044	0.158	1.485	−0.209	3.615
gov	2856	0.934	0.579	0.112	0.752	6.071	2.979	33.115
fin	2856	0.003	0.003	0.000	0.002	0.030	2.216	10.399
fdi	2856	0.481	0.099	0.117	0.482	0.851	2.032	11.309
∅ lngdp	2856	16.552	0.918	14.067	16.452	19.760	0.464	3.238
∅ lnpop	2856	14.723	0.831	12.387	14.694	18.241	0.579	4.307
∅ lngov	2856	5.965	0.641	3.833	5.986	8.134	−0.358	3.392
∅ lnind	2856	15.796	0.946	12.863	15.754	18.469	0.120	3.008

## 4 Results

### 4.1 Measurement of urban carbon emissions performance

Table 3 reports the estimation results of the stochastic frontier model. The estimated coefficients of ∅ lngdp, ∅ lnpop, ∅ lnge and ∅ lnind are 0.116, 0.061, 0.128 and 0.089 respectively, all passing the 1 % significance level test. Based on the study of Kumbhakar et al. [39], this paper separates the time-varying and time-invariant features and individual heterogeneity from the residuals of the estimation results and captures the urban carbon emissions performance.

**Table 3:** Estimated results of stochastic frontier model.

	Coefficient	T-value
***Basic Regression***		
∅ lngdp	0.116***	9.141
∅ lnpop	0.061***	2.653
∅ lnge	0.128***	12.984
∅ lnind	0.089***	6.720
*constant*	13.100***	87.288
*Frontier constant*	0.064***	31.202
***Inefficiency and error term***		
**C**_***u***_	−5.002***	−83.177
**C**_***v***_	−6.292***	−102.254
Log likelihood	941.21	

Note***, **, and * denote significant at the 1 % level, 5 % level and 10 % level. ***C***_***u***_ denotes the unconstrained parameters in the parameterization equation exp(***C***_***u***_) = $\sigma_{u}^{2}$. ***C***_***v***_ denotes the unconstrained parameters in the parameterization equation exp(***C***_***ν***_) = $\sigma_{v}$.

**Fig. 1:**
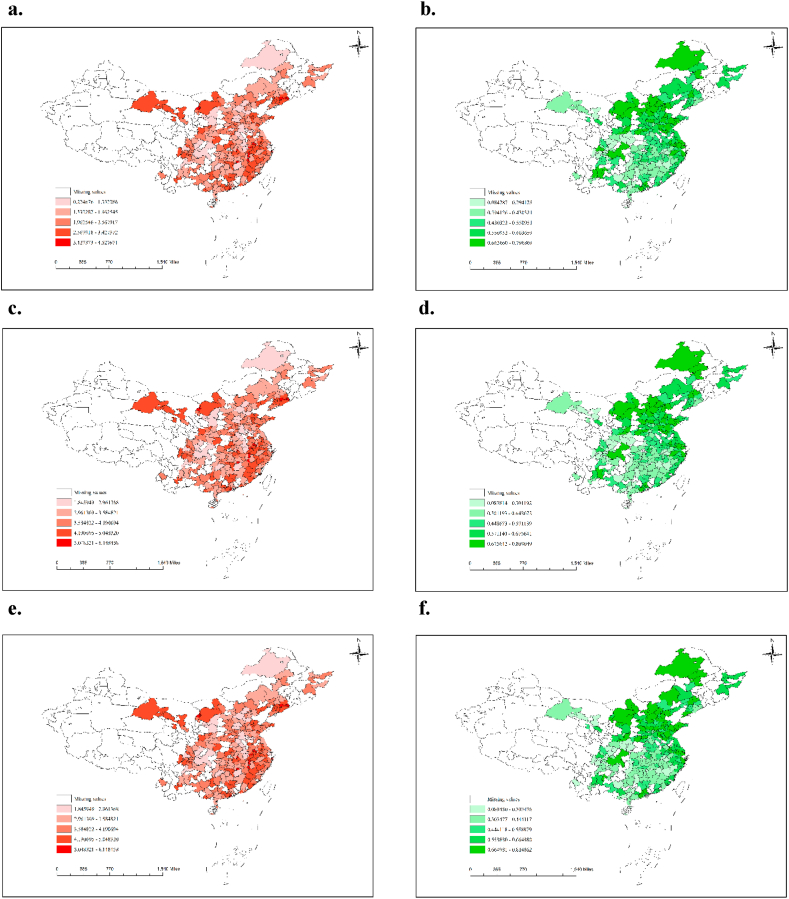
Distribution of IINT and CEP.1.

The right panel of Fig. 1 shows the urban CEP under SFA method in 2010, 2015 and 2019. The map is color-coded, with darker colors indicating better CEP. The results reveal that cities in the northern region have higher average CEP than those in the southern region across the three years examined. Additionally, coastal cities exhibit better CEP than their inland counterparts located at the same latitude. Moreover, the study shows that CEP in China displays a relatively stable trend from 2010 to 2019, with a general upward trend over this period. Fig. 1 also illustrates the distribution of industrial intelligence across China in 2010, 2015, and 2019. The left section of the figure shows a color-coded map of industrial intelligence, with darker colors indicating higher usage rates. The analysis reveals that the eastern coastal region has a higher concentration of industrial intelligence installations, while the inland regions have fewer installations. The study also finds that there is no significant difference in industrial intelligence usage between the northern and southern regions. Furthermore, there has been a significant increase in industrial robot usage in China from 2010 to 2019, indicating an overall upward trend.

### 4.2 Spatial autocorrelation test

**Fig. 2:**
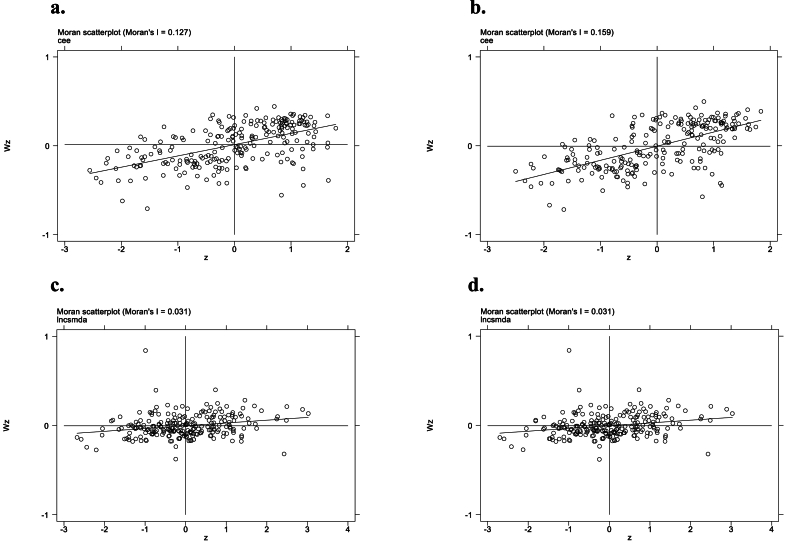
Moran scatter plot of CEP and industrial intelligence.^2^.

Our study investigates spatial autocorrelation of CEP in Chinese cities and uses the Moran scatter plot to visualize the pattern. The scatter plots in Fig. 2 (upper section) illustrate a positive spatial correlation among urban CEP. The left and right plots display the standardized CEP and spatial lag value, respectively, for each city in 2010 and 2019. The coefficients of the primary fit lines are significantly greater than 0, indicating a positive association among urban CEP.

The Moran index of CEP results presented in Table 4 indicate a significant positive spatial correlation between 2008 and 2019. These findings demonstrate a strong spatial association in CEP among Chinese cities. Furthermore, this study evaluates the spatial correlation of industrial intelligence usage using Moran index scatter plots. The lower portion of Fig. 2 shows scatter plots of industrial intelligence usage for all cities in the year of 2010 and year of 2019, respectively. X represents standardized industrial intelligence usage, while Y represents spatial lag values. Coefficients of fit lines are all significantly greater than 0, suggesting a positive spatial correlation among industrial intelligence. The Moran index analysis results presented in Table 4 indicate a strong spatial correlation in the usage of industrial intelligence in Chinese cities between 2008 and 2019. Therefore, it is crucial to consider the spatial factor when estimating the model to ensure the validity and reliability of the results.

**Table 4:** Moran's I index computation.

Year	*CEP*		*IINT*	
	Moran's I	Z-value	Moran's I	Z-value
2008	0.127**	15.231	0.031***	4.125
2009	0.131***	15.658	0.031***	4.106
2010	0.138***	16.533	0.031***	4.127
2011	0.141***	16.917	0.031***	4.120
2012	0.142***	17.032	0.031***	4.113
2013	0.143***	17.146	0.031***	4.111
2014	0.145***	17.367	0.031***	4.129
2015	0.148***	17.661	0.031***	4.121
2016	0.152***	18.112	0.031***	4.110
2017	0.155***	18.430	0.031***	4.112
2018	0.154***	18.418	0.031***	4.081
2019	0.159***	18.937	0.031***	4.095

Note: ***, **, and * denote significant at the 1 % level, 5 % level and 10 % level.

### 4.3 Baseline results

Table 5 displays the outcomes of various regression models. Fixed effects method, SLM method, SEM method, and SDM method were employed, and impact of industrial intelligence on CEP was found to be statistically significant across all models. The coefficients obtained from the four models show that 1 % rise in industrial intelligence will contribute to an increase in CEP by 0.091 (p < 0.01), 0.063 (p < 0.01), 0.072 (p < 0.01), and 2.747 (p < 0.01), respectively. SDM method was found to be the most robust due to its ability to account for spatial lag and spatial error effects. The results from the SDM method showed that a 1 % improvement in industrial intelligence leads to a 2.747 % enhancement in CEP.

**Table 5:** Spatial spillover effect of the industrial intelligence on urban CEP.

Variables	FE	SLM	SEM	SDM
	(1)	(2)	(3)	(4)
***IINT***	0.091***	0.063***	0.072***	2.747***
	(0.008)	(0.007)	(0.010)	(0.826)
ln***rgdp***	−0.220***	−0.229***	−0.252***	−0.178***
	(0.023)	(0.021)	(0.023)	(0.023)
***is***	0.832***	0.339***	−0.148	−0.361***
	(0.099)	(0.092)	(0.107)	(0.101)
***gov***	0.435***	0.315**	0.144	0.031
	(0.141)	(0.125)	(0.126)	(0.124)
***fin***	0.053**	0.084***	0.113***	0.114***
	(0.024)	(0.022)	(0.021)	(0.021)
***fdi***	1.896	2.645	3.838*	0.088
	(2.428)	(2.156)	(2.050)	(2.042)
***wIINT***				−2.733***
				(0.826)
***w***ln***rgdp***				0.308***
				(0.037)
***wis***				1.843***
				(0.183)
***wgov***				0.345
				(0.258)
***wfin***				−0.146***
				(0.048)
***wfdi***				−18.794***
				(4.413)
City FE	Y	Y	Y	Y
Year FE	Y	Y	Y	Y
Observation	2760	2760	2760	2760
Log-L	6329.407	7657.811	6205.237	7372.020
R^2^	0.098	0.244	0.203	0.277

note: (1) ***, **, and * indicate significance at the 1 %, 5 %, and 10 % levels, respectively. (2) The reported standard errors are cluster-robust at the city-level and are shown in parentheses. (3) City FE and Year FE refer to the city fixed effects and year fixed effects, respectively. (4) The following tables in this paper also apply this note.

Moreover, this study investigates the influence of economic development on the environment. Our study found that per capita GDP's effect on CEP was negative at the 1 % level of significance. The impact of industrial structure on CEP varied in different models. The fixed-effects model and SLM method demonstrated that industrial structure has a positive effect on CEP at the 1 % level of significance, while the results of SEM method were not significant, and the SDM method showed a negative effect on CEP at the 1 % level of significance. Government governance was found to enhance carbon performance at the 1 % and 5 % level of significance in the fixed-effects model and SLM method, respectively. Financial development was found to increase CEP at the 5 % level of significance. Finally, the effect of FDI on CEP was not significant, except for the SEM method, which showed a positive effect at the 10 % level of significance. The baseline regression using the SDM method reveals a positive impact of industrial intelligence on CEP. Since industrial intelligence is distributed throughout the country and has spatial correlation, discussing the spatial spillover effect of industrial intelligence on CEP is necessary.

Table 6 shows the spatial spillover effect of industrial intelligence on CEP. The direct effect indicates the effect on local CEP, the indirect effect indicates the effect on neighboring CEP and the total effect combines the effects of direct and indirect effects. Specifically, the direct effect of industrial intelligence on carbon performance is positive at 1 % level of significance, and the indirect effect of industrial intelligence on carbon performance is negative at 1 % level of significance. The total effect of industrial intelligence on carbon performance is positive at 1 % level of significance. It shows that industrial intelligence has an enhancing effect on the carbon performance in the local region, but a decreasing effect on the carbon performance in neighboring regions. Studies by Liu et al. [26] and Li et al. [27] have demonstrated that industrial intelligence has a significant contribution to CEP. However, their papers do not consider the spatial spillover effects. Our paper shows that the direct effect of industrial intelligence is effective in improving CEP, but the indirect effect worsens it.

**Table 6:** Direct, indirect, and total effects of SDM in Table 5.

	*IINT*	ln*rgdp*	*is*	*gov*	*fin*	*fdi*
Direct effect	2.686***	−0.166***	−0.262***	0.048	0.109***	−0.739
	(0.819)	(0.022)	(0.096)	(0.123)	(0.021)	(2.061)
Indirect effect	−2.665***	0.363***	2.543***	0.540	−0.154**	−27.304***
	(0.818)	(0.051)	(0.244)	(0.366)	(0.074)	(6.623)
Total effect	0.022***	0.197***	2.281***	0.588	−0.045	−28.043***
	(0.004)	(0.052)	(0.253)	(0.400)	(0.083)	(7.455)

note: (1) ***, **, and * indicate significance at the 1 %, 5 %, and 10 % levels, respectively. (2) The reported standard errors are cluster-robust at the city-level and are shown in parentheses. (3) City FE and Year FE refer to the city fixed effects and year fixed effects, respectively. (4) The following tables in this paper also apply this note.

By combing the existing literature, this paper argues that the reason leading to the difference between the impact of industrial intelligence on local carbon emission performance and that of neighboring regions lies in the evolution of industrial structure. In the hypothesis section of the study, the paper points out that a key factor lies in the clustering of productive services. It has been shown in the literature that industrial intelligence has a significant positive impact on the agglomeration of productive services, and that the agglomeration of productive services also significantly leads to improved carbon emission performance [64,67]. Considering the spatial correlation, the increase of local industrial intelligence will lead to the increase of local productive service industry agglomeration and the decrease of the level in the neighboring areas. As a result, industrial intelligence enhances the local carbon emission performance but suppresses the carbon emission performance of neighboring regions. The paper empirically tests the above mechanisms through a mediated effects model in Section 6. The new findings of this paper can provide suggestions for governments in developing cross-regional carbon reduction policies, and it can also provide further support for sustainable development issues related to industrial intelligence.

### 4.4 Robustness test

#### 4.4.1 Re-estimation using different dependent variable

To mitigate the potential influence of variable settings on the regression outcomes, this study adopts an alternative approach for measuring the independent variable. Instead of using an enhanced SFA model employed previously, this study measures CEP as the ratio of carbon emissions to GDP.

Table 7 presents the regression results obtained using this revised dependent variable measure and examines the effect of industrial intelligence on CEP. Across the fixed effects, SLM, SEM, and SDM methods, the results indicate that industrial intelligence positively impacts CEP by 7.1 %, 6.0 %, 10 %, and 137.7 %, respectively, all of which pass the significance test at the 1 % level. The results of the regression model using the revised measure of the dependent variable are consistent with those presented in the previous section, indicating the reliability of our findings.

**Table 7:** Re-estimation based on another dependent variable.

Variables	FE	SLM	SEM	SDM
	(1)	(2)	(3)	(4)
***IINT***	0.071***	0.060***	0.100***	1.377***
	(0.004)	(0.005)	(0.006)	(0.476)
ln***rgdp***	0.482***	0.471***	0.434***	0.338***
	(0.013)	(0.013)	(0.015)	(0.013)
***is***	−0.185***	−0.139**	0.231***	0.665***
	(0.056)	(0.055)	(0.080)	(0.058)
***gov***	−0.203**	−0.242***	−0.320***	−0.314***
	(0.080)	(0.077)	(0.078)	(0.071)
***fin***	−0.054***	−0.060***	−0.094***	−0.107***
	(0.014)	(0.013)	(0.014)	(0.012)
***fdi***	−0.801	−0.752	−0.437	1.862
	(1.371)	(1.308)	(1.272)	(1.176)
***wIINT***				−1.424***
				(0.476)
***w***ln***rgdp***				0.091***
				(0.027)
***wis***				−1.382***
				(0.103)
***wgov***				0.598***
				(0.148)
***wfin***				0.162***
				(0.028)
***wfdi***				4.485*
				(2.543)
City FE	Y	Y	Y	Y
Year FE	Y	Y	Y	Y
Observation	2760	2760	2760	2760
R^2^	0.143	0.268	0.263	0.292

note: (1) ***, **, and * indicate significance at the 1 %, 5 %, and 10 % levels, respectively. (2) The reported standard errors are cluster-robust at the city-level and are shown in parentheses. (3) City FE and Year FE refer to the city fixed effects and year fixed effects, respectively. (4) The following tables in this paper also apply this note.

#### 4.4.2 Re-estimation based on different independent variable

This study conducts a robustness test of the regression analysis by substituting different independent variables. Specifically, the previous section measured the level of industrial intelligence using the number of uses of industrial robots, while in this section, the level of industrial intelligence is measured by the number of owned industrial robots.

Table 8 presents the results of the regression analysis after replacing the independent variables, focusing on the effect of industrial intelligence on CEP. The fixed effects method, SLM method, SEM method, and SDM method demonstrate that industrial intelligence has significantly positive impacts on CEP, with increases of 6.2 %, 4.5 %, 4.9 %, and 478.9 %, respectively, and all passing the significance test at 1 %. The robustness check of substituting independent variables indicates the stability of the findings in this study and confirms the reliability of the previous results.

**Table 8:** Re-estimation based on different independent variable.

Variables	FE	SLM	SEM	SDM
	(1)	(2)	(3)	(4)
***OIR***	0.062***	0.045***	0.049***	4.788***
	(0.010)	(0.009)	(0.010)	(1.294)
ln***rgdp***	−0.151***	−0.190***	−0.220***	−0.177***
	(0.029)	(0.025)	(0.024)	(0.024)
***is***	0.878***	0.353***	−0.246**	−0.382***
	(0.111)	(0.101)	(0.109)	(0.102)
***gov***	0.605***	0.406***	0.209*	0.019
	(0.147)	(0.130)	(0.127)	(0.125)
***fin***	0.059**	0.088***	0.122***	0.112***
	(0.025)	(0.022)	(0.021)	(0.021)
***fdi***	1.009	2.200	3.494*	0.353
	(2.485)	(2.182)	(2.050)	(2.044)
***wOIR***				−4.780***
				(1.294)
***w***ln***rgdp***				0.332***
				(0.050)
***wis***				1.872***
				(0.196)
***wgov***				0.346
				(0.262)
***wfin***				−0.150***
				(0.050)
***wfdi***				−20.833***
				(4.452)
City FE	Y	Y	Y	Y
Year FE	Y	Y	Y	Y
Observation	2760	2760	2760	2760
R^2^	0.063	0.232	0.159	0.288

note: (1) ***, **, and * indicate significance at the 1 %, 5 %, and 10 % levels, respectively. (2) The reported standard errors are cluster-robust at the city-level and are shown in parentheses. (3) City FE and Year FE refer to the city fixed effects and year fixed effects, respectively. (4) The following tables in this paper also apply this note.

#### 4.4.3 Re-estimation based on different spatial weight matrix

In addition to replacing the dependent and independent variables, this paper also uses the method of replacing the spatial weight matrix to conduct robustness test. In the previous section, we used geographic distance weight matrix in the spatial panel model. Here, this paper uses the economic distance weight matrix as the spatial weight matrix. Table 9 reports the effect of industrial intelligence on CEP based on the geographic distance weight matrix (W1) and the economic distance weight matrix (W2). When the geographic distance weight matrix is used as the spatial weight matrix, industrial intelligence can contribute to 3.7 %, 2.9 % and 213.6 % of CEP at 1 % significance level under the SLM method, SEM method and SDM method, respectively. When the economic distance weight matrix is used as the spatial weight matrix, industrial intelligence can contribute to 3.2 %, 19.3 % and 226.3 % of the CEP at 1 % level of significance under the SLM method, SEM method and SDM method, respectively. The outcome suggest that the regression analysis remains consistent with the previous study after replacing the spatial weight matrix. This indicates the robustness of the previous findings.

**Table 9:** Re-estimation based on different spatial weight matrix.

Variables	*W1*			*W2*		
	SLM	SEM	SDM	SLM	SEM	SDM
	(1)	(2)	(3)	(4)	(5)	(6)
***IINT***	0.037***	0.029***	2.136***	0.032***	0.193***	2.263***
	(0.007)	(0.009)	(0.749)	(0.006)	(0.070)	(0.746)
ln***rgdp***	−0.247***	−0.230***	−0.119***	−0.155***	−0.150***	−0.123***
	(0.019)	(0.022)	(0.022)	(0.017)	(0.022)	(0.022)
***is***	−0.235***	−0.670***	−0.392***	−0.231***	−0.485***	−0.509***
	(0.087)	(0.099)	(0.094)	(0.072)	(0.092)	(0.092)
***gov***	0.098	−0.001	−0.012	0.102	0.054	−0.052
	(0.118)	(0.118)	(0.113)	(0.101)	(0.109)	(0.110)
***fin***	0.111***	0.141***	0.084***	0.060***	0.067***	0.039**
	(0.020)	(0.020)	(0.019)	(0.017)	(0.019)	(0.019)
***fdi***	1.804	1.813	−1.685	1.816	1.599	2.599
	(2.018)	(1.949)	(1.873)	(1.744)	(1.822)	(1.825)
***wIINT***			−2.270***			−2.230***
			(0.749)			(0.747)
***w***ln***rgdp***			1.220***			−0.119**
			(0.072)			(0.052)
***wis***			5.254***			1.575***
			(0.454)			(0.258)
***wgov***			−7.454***			0.941
			(1.057)			(0.600)
***wfin***			0.579***			0.320***
			(0.150)			(0.099)
***wfdi***			−181.519***			−6.784
			(20.329)			(11.629)
City FE	Y	Y	Y	Y	Y	Y
Year FE	Y	Y	Y	Y	Y	Y
Observation	2760	2760	2760	2760	2760	2760
R^2^	0.087	0.099	0.134	0.120	0.114	0.185

note: (1) ***, **, and * indicate significance at the 1 %, 5 %, and 10 % levels, respectively. (2) The reported standard errors are cluster-robust at the city-level and are shown in parentheses. (3) City FE and Year FE refer to the city fixed effects and year fixed effects, respectively. (4) The following tables in this paper also apply this note.

### 4.5 Endogeneity test

The previous section used spatial panel regression to explore the impact of industrial intelligence on CEP and obtained positive results. However, endogeneity cannot be eliminated by using spatial measures, which challenges the robustness of the regression results. This paper treats the implementation of Industry 4.0 policy as a quasi-natural experiment and re-estimates it based on DID and PSM-DID methods. The results presented in Table 10 show that the implementation of Industry 4.0 policies promotes CEP at 1 % level of significance under both DID and PSM-DID approaches. The present findings using spatial measures are consistent with previous research, suggesting that the original regression model has passed the endogeneity test. Therefore, the results of the original regression are robust.

**Table 10:** Endogeneity test based on DID estimation.

Variables	*DID*	*PSM-DID*
	(1)	(2)
***dudt***	0.173***	0.283***
	(0.015)	(0.020)
***C***	5.441***	7.647***
	(0.136)	(0.195)
Control	Y	Y
City FE	Y	Y
Year FE	Y	Y
Observation	2760	1616
Log-L	47.926	60.026
R^2^	0.087	0.178

note: (1) ***, **, and * indicate significance at the 1 %, 5 %, and 10 % levels, respectively. (2) The reported standard errors are cluster-robust at the city-level and are shown in parentheses. (3) City FE and Year FE refer to the city fixed effects and year fixed effects, respectively. (4) The following tables in this paper also apply this note.

## 5 Heterogeneity analysis

Compared with regions with a lower level of economic development, regions with a higher degree of economic development have a more complete industrial system, and their industrial intelligence enhancement has a greater impact on the input and output of production factors [82]. Therefore, it is necessary to carry out a heterogeneity analysis from the perspective of the level of economic development. There are huge regional inequalities in China's economic development [83]. Specifically, the economic development level in the eastern region of China is significantly higher than that in the central and western regions at present [84]. Thus, this paper explores the heterogeneity of the impact of industrial intelligence on CEP in the eastern, central and western regions of China. Then, resource-dependent cities accomplish intelligence conversion at the industrial level more easily than non-resource-dependent cities [85]. Hence, our study also discusses impact of industrial intelligence on CEP in different resource-dependent cities. Finally, heterogeneity analysis also needs to consider the administrative rank of the city. The administrative level of cities plays an important role in the allocation of resources in China [86]. Cities with higher administrative level have higher access to resources [87]. This indicates that high administrative level cities are more attractive to firms with high industrialization level and CEP than lower administrative level cities. Therefore, this paper discusses the effect of industrial intelligence on CEP in different administrative-level cities.

The results of the heterogeneity analysis of the impact of industrial intelligence on CEP are presented in Table 11. Row (1) of Table 11 shows the baseline regression results for the impact of industrial intelligence on CEP in eastern cities, non-resource-dependent cities, and general prefecture-level cities, respectively. The impact of industrial intelligence on CEP in eastern cities is positive and statistically significant at the 1 % level of significance, with a coefficient of 2.314, reflecting the favorable geographical location of the eastern region. The impact of industrial intelligence on CEP in central cities is lower than that in eastern cities, but still statistically significant at the 1 % level of significance. In contrast, the effect of industrial intelligence on CEP in western cities is not significantly different from that in eastern cities. These findings suggest that the government should prioritize the role of industrial intelligence in enhancing CEP in the central region, while also considering the potential benefits of industrial intelligence for CEP in the eastern and western regions. Thus, it will promote the synergistic development of industrial transformation to environment-friendly enterprises in eastern, central and western China. The degree of resource dependence of the city also affects the effect of industrial intelligence on CEP. For non-resource-dependent cities, the coefficient of the effect of industrial intelligence on CEP is 3.126 and is positive at 1 % level of significance. The effect of industrial intelligence on CEP in resource-dependent cities is higher than that in non-resource-dependent cities at 1 % significance level, with an increase of 0.052. This indicates that the country should promote the level of technological innovation in non-resource-dependent cities to further enhance the effectiveness of industrial intelligence on CEP in non-resource-dependent cities. This paper also explores the effect of city administrative level on industrial intelligence driving CEP. The coefficient of the effect of industrial intelligence on CEP is 2.626 in the ordinary prefecture-level cities, and it is positive at 1 % level of significance. In contrast, the coefficient of the effect of industrial intelligence on CEP is reduced by 0.024 in other high administrative-level cities, and this effect is negative at 1 % level of significance level. It shows that government should focus more on important utility of industrial intelligence in central cities for green development, and effectively improve the environmental performance of the whole region.

**Table 11:** Heterogeneity analysis.

Variables	Region	Resource dependency	Administrative rank
	(1)	(2)	(3)
***IINT***	2.314***	3.126***	2.626***
	(0.823)	(0.812)	(0.819)
***IINT × central***	−0.020***		
	(0.006)		
***IINT × western***	−0.001		
	(0.007)		
***IINT × res***		0.052***	
		(0.005)	
***IINT × rank***			−0.024***
			(0.008)
ln***rgdp***	−0.174***	−0.131***	−0.164***
	(0.023)	(0.023)	(0.023)
***is***	−0.347***	−0.197*	−0.343***
	(0.100)	(0.101)	(0.100)
***gov***	0.174	0.184	0.045
	(0.124)	(0.123)	(0.124)
***fin***	0.102***	0.135***	0.134***
	(0.021)	(0.020)	(0.021)
***fdi***	3.183	0.944	1.167
	(2.115)	(2.010)	(2.035)
***wIINT***	−2.254***	−3.144***	−2.631***
	(0.823)	(0.812)	(0.819)
***wIINT × central***	−0.049***		
	(0.015)		
***wIINT × western***	−0.117***		
	(0.017)		
***wIINT × res***		0.001	
		(0.012)	
***wIINT × rank***			0.126***
			(0.017)
***w***ln***rgdp***	0.367***	0.275***	0.309***
	(0.038)	(0.038)	(0.037)
***wis***	1.873***	1.741***	1.819***
	(0.183)	(0.185)	(0.181)
***wgov***	0.336	0.621**	0.862***
	(0.262)	(0.262)	(0.267)
***wfin***	−0.146***	−0.186***	−0.218***
	(0.048)	(0.048)	(0.049)
***wfdi***	−12.868***	−15.220***	−13.816***
	(4.601)	(4.360)	(4.451)
City FE	Y	Y	Y
Year FE	Y	Y	Y
Observation	2760	2760	2760
R^2^	0.302	0.304	0.292

note: (1) ***, **, and * indicate significance at the 1 %, 5 %, and 10 % levels, respectively. (2) The reported standard errors are cluster-robust at the city-level and are shown in parentheses. (3) City FE and Year FE refer to the city fixed effects and year fixed effects, respectively. (4) The following tables in this paper also apply this note.

The above heterogeneity analysis explores the impact of industrial intelligence on CEP from the perspective of urban location, resource dependency and administrative level of cities, respectively. Wang et al. [47] showed that CEP in China decreased spatially in the east, central, and west using data from 2005 to 2016. Fan et al. [88] explored the relationship between resource dependence and CEP using provincial panel data in China and pointed out the imbalance in carbon emission reduction efficiency among cities with different resource dependence. Based on the previous study, our results suggest that policy makers need to ensure that carbon reduction efforts in the east, middle and west are balanced. On the other hand, they should pay equal attention to the sustainable development of cities with different resource dependence. Meanwhile, policy makers should make more use of the role of central cities to improve their CEP and spread it to the surrounding areas.

## 6 Mechanism analysis

The findings in the previous section suggest that industrial intelligence enhances local CEP but reduces CEP in surrounding areas. In section 2.3, this paper proposes the research hypothesis that the reason for the above findings is that industrial intelligence contributes to the development of producer services, while the local agglomeration of producer services enhances local CEP but reduces CEP in neighboring areas. Table 12 reports the result of the mechanism test for the agglomeration of producer services.

**Table 12:** Mechanism test based on producer services agglomeration.

	AGG		CEP	
Variables	Direct effect	Indirect effect	Direct effect	Indirect effect
	(1)	(2)	(3)	(4)
***IINT***	3.682***	−3.671***	3.558***	−3.542***
	(0.512)	(0.512)	(0.823)	(0.823)
***agg***			0.237***	0.162***
			(0.031)	(0.029)
ln***rgdp***			−0.148***	0.389***
			(0.022)	(0.050)
***is***			−0.225**	2.447***
			(0.093)	(0.239)
***gov***			−0.013	0.581
			(0.121)	(0.377)
***fin***			0.104***	−0.163**
			(0.021)	(0.074)
***fdi***			−1.322	−27.610***
			(2.133)	(6.435)
City FE	Y	Y	Y	Y
Year FE	Y	Y	Y	Y
Observation	2760	2760	2760	2760
Log-L	2385.887	2385.887	1043.893	1043.893
R^2^	0.084	0.084	0.291	0.291

note: (1) ***, **, and * indicate significance at the 1 %, 5 %, and 10 % levels, respectively. (2) The reported standard errors are cluster-robust at the city-level and are shown in parentheses. (3) City FE and Year FE refer to the city fixed effects and year fixed effects, respectively. (4) The following tables in this paper also apply this note.

In Table 12, the results of regressions (1) and (2) show that the direct effect of industrial intelligence on producer services agglomeration is positive at 1 % level of significance with a coefficient of 3.682. While the indirect effect of industrial intelligence on the agglomeration of producer services is negative at 1 % level of significance with a coefficient of −3.671. The results of regressions (3) and (4) show that both the direct and indirect effects of the impact of producer services agglomeration on CEP are positive at 1 % level of significance, and the coefficients are 0.237 and 0.162, respectively. In terms of the agglomeration of producer service industries on CEP, the finding of this paper is consistent with Ma and Yao [89], which suggests that the increase in the agglomeration level of producer service industries can eventually improve the carbon efficiency in both local and neighboring regions. However, the above mechanism analysis further shows that industrial intelligence increases the local producer service agglomeration and decreases the producer service agglomeration in neighboring areas. It means that industrial intelligence will improve local CEP by promoting local producer service agglomeration but will reduce CEP in neighboring areas by reducing producer service agglomeration. The findings explain the mechanism of industrial smartness affecting CEP and suggest that promoting industrial intelligence improves local CEP but inhibits carbon emission mitigation in neighboring regions. This poses a new challenge for industrial and climate policy making, and governments should comprehensively assess the emission reduction effects of industrial intelligence to balance efficiency and equity.

## 7 Conclusion and recommendations

Based on the balanced panel data of 238 cities from 2008 to 2019 in China, this paper investigates the effect of industrial intelligence on CEP. Meanwhile, this paper conducts a more in-depth study of the spatial spillover effects and mechanisms of industrial intelligence on CEP, and test the robustness of our results. Our research indicates that a 1 % improvement in industrial intelligence leads to a 2.747 % enhancement of CEP. When considering the spatial spillover effect, the direct, indirect and total effects of industrial intelligence on CEP are 2.686 %, −2.665 % and 0.022 %, respectively. In addition, this paper explains the mechanism of industrial intelligence affecting CEP at the spatial level from the perspective that industrial intelligence affects the concentration of producer service industries in local and neighboring areas and thus affects CEP. Our results illustrate that industrial intelligence can effectively enhance the local CEP, but also significantly reduce CEP of neighboring regions. Further heterogeneity tests illustrate that the enhancement effect of industrial intelligence on CEP is more pronounced in eastern city, ordinary prefecture-level city and resource-dependent city. Finally, the endogeneity and robustness tests produced consistent findings, providing further support for the results. The scientific value of this paper lies in revealing the spatial spillover effects of industrial intelligence on urban carbon emission performance and the net effect after considering spatial correlations. Most of the existing studies show that industrial intelligence can effectively promote productivity gains and has positive spatial spillover effects. However, the relationship between industrial intelligence and urban carbon emission performance has not been fully investigated, as industrial intelligence also brings about drastic changes in production methods, which will inevitably affect the urban carbon emissions performance. This paper examines the impact of industrial intelligence on the urban carbon emission performance through panel data of Chinese cities, which can provide a theoretical basis for the formulation of policies on climate change and the development of artificial intelligence.

Drawing on the results, three recommendations can be suggested. Firstly, it is suggested that the government should develop a more comprehensive policy framework to promote the adoption and diffusion of industrial intelligence technologies. Current industrial intelligence can effectively improve the local CEP, but it will harm the CEP of the surrounding areas. A healthy industrial intelligence policy can not only ensure the sustainable development of the region, but also positively influence the neighboring regions through spillover effects, and thereby play a synergistic role in improving the regional CEP. Secondly, the government should emphasize the moderating role of producer service agglomeration in the impact of industrial intelligence on CEP. The government should refer to the path of industrial intelligence to improve or reduce CEP, and reasonably adjust the level of producer service agglomeration based on the level of intelligence. The synergistic development of industrial intelligence and producer service industries can improve CEP and contribute to sustainable development in a more effective way. Finally, the government should consider the city's location, resources and hierarchical characteristics when formulating industrial intelligence policies. Our results show that industrial intelligence has a relatively weak effect on improving CEP in central regions, high administrative-level cities, and non-resource-dependent cities. Therefore, the government should focus on enhancing the effectiveness of industrial intelligence in promoting CEP in cities with these characteristics. Ultimately, society needs to achieve a higher level of industrial intelligence and induce its comprehensive, deep and sustained improvement on CEP.

## Data availability statement

3rd Party Data. Restrictions apply to the availability of these data. Data was obtained from National Bureau of Statistics in China and are available with the per-mission of Guangzhou University.

## CRediT authorship contribution statement

**Chenglin Tu:** Writing – review & editing, Writing – original draft, Methodology, Data curation. **Chuanxiang Zang:** Writing – original draft, Formal analysis, Data curation. **Anqi Wu:** Writing – review & editing. **Hongyu Long:** Writing – original draft, Formal analysis, Data curation. **Chenyang Yu:** Writing – review & editing, Methodology, Funding acquisition, Data curation. **Yuqing Liu:** Writing – review & editing, Resources, Funding acquisition, Conceptualization.

## Declaration of competing interest

The authors declare that they have no known competing financial interests or personal relationships that could have appeared to influence the work reported in this paper.

## Footnotes

1 The left-side panel **a**, **c** and **e** show the distribution of IINT nationwide in 2010, 2015 and 2019. The right-side panel **b**, **d** and **f** show the graphs of urban CEP under SFA method in 2010, 2015 and 2019.

2 The panel **a** and **b** show the scatter plots of CEP for each city in 2010 and 2019. The panel **c** and **d** show scatter plots of all cities' industrial intelligence in 2010 and 2019.
